# Designing Safer Transitions: Proactively Reducing Ambulatory Staff Harm from Patient Behavioral Events

**DOI:** 10.1097/pq9.0000000000000827

**Published:** 2025-08-29

**Authors:** Laura R. Nicholson, Laurel P. Scarfo

**Affiliations:** From the Quality, Safety and Performance, Holland Bloorview Kids Rehabilitation Hospital, Toronto, Ontario, Canada.

## Introduction:

Holland Bloorview Kids Rehabilitation Hospital supports clients with complex behavioral and developmental needs, where transitions to and from ambulatory care can pose safety risks for staff. To proactively address these challenges, the Walk-Through Talk-Through (WT3) tool, encouraged by Solutions for Patient Safety, was implemented. This structured tool was used to identify and address behavioral safety risks to support safer transitions in care. Grounded in a “work-as-done” perspective, WT3 explored how transitions occur in practice, rather than solely as intended. By capturing frontline workflows, improvisations, and system pressures, WT3 revealed human factors and latent safety threats. These insights informed codesigned protocols and the planning of a new clinical space in the Extensive Needs Services (ENS) program.

## Methods:

WT3 involved frontline staff, clients, and caregivers in observing and mapping transition touchpoints, including arrivals, movement between spaces, and postvisit exits. WT3 supports staff in identifying proactive safety measures. Perspectives from clients and caregivers were also gathered to assess necessary precautions. Potential risks, such as miscommunication, client escalation, and environmental hazards, were identified. Solutions and mitigation strategies were codesigned with ENS staff, security, and program leaders. Mitigation strategies were codesigned with ENS clinicians, security staff, and leadership and incorporated into both care protocols and physical space design.

The goal is to ensure the safety and well-being of Holland Bloorview staff while also fostering an inclusive environment that supports the integration of clients with high behavioral needs. We are committed to minimizing staff harm through appropriate strategies, training, and support, in addition to environmental design. This ensures that we promote respectful, person-centered care that upholds the dignity and potential of every individual with a focus on the principle of least restraint.

With the new clinical space for ENS now built, there will be ongoing assessment of the impact of WT3 using key metrics, such as staff harm data, family satisfaction, safety event reporting, and patient behavioral events data.

## Results:

Risks identified included missed or unclear registration, escalations in unsecured areas, overstimulating environments, and infrastructure issues. Mitigation strategies implemented include:

- designated client entry points and improved elevator access;
- secured clinic doors with badge access to prevent elopement;
- environmental modifications to reduce sensory triggers;
- access to preferred items to support regulation.

**Fig. 1.**
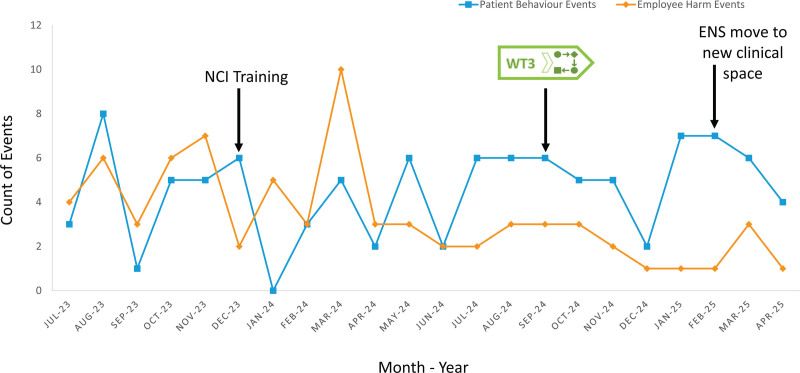
Patient behavior events vs employee harm events related to patient behavior events. A spike in employee harm occurred in March 2024 despite prior crisis training. After WT3 implementation in September 2024, employee harm events declined and remained low, even as behavioral events continued. This suggests that WT3 effectively reduced staff harm during transitions. NCI, nonviolent crisis intervention.

## Conclusions:

WT3 enabled the early identification of behavioral safety risks and supported practical, codesigned solutions. Integrating these strategies into workflows and infrastructure improved safety for clients, families, and staff (see Fig. 1 ). This model demonstrates the value of proactive design thinking and cross-disciplinary collaboration in creating safer pediatric ambulatory environments.

## ACKNOWLEDGMENTS

The authors gratefully acknowledge the contributions of the Holland Bloorview ENS team and the Solutions for Patient Safety Network for their support and collaboration in this work.

